# Activismos locos en salud mental: una revisión integrativa

**DOI:** 10.18294/sc.2023.4627

**Published:** 2023-11-28

**Authors:** Juan Carlos Cea Madrid

**Affiliations:** 1 Psicólogo. Magíster en psicología, mención psicología comunitaria. Estudiante de Doctorado en Psicología. Universidad de Chile, Santiago, Chile. jcarlos.ceamadrid@gmail.com Universidad de Chile Universidad de Chile Santiago Chile jcarlos.ceamadrid@gmail.com

**Keywords:** Salud Mental, Activismo Político, Derechos Humanos, Justicia Social, Mental Health, Political Activism, Human Rights, Social Justice

## Abstract

Se exponen los resultados de una revisión integrativa que tuvo como propósito identificar y analizar la producción de literatura académica sobre el activismo loco en el campo de la salud mental y su vinculación con las nociones de discapacidad y neurodiversidad. De las búsquedas realizadas en mayo del 2023, en las bases de datos Web of Science, Scopus y PubMed, se seleccionaron 52 artículos, y se aplicaron estrategias de análisis de contenido temático. Los resultados dan cuenta de diversas articulaciones entre los conceptos abordados, estableciendo una mirada crítica hacia el modelo biomédico en salud mental. En las formas de activismo loco, adquieren relevancia el enfoque de derechos humanos, la lucha contra el estigma y su influencia en los procesos de reforma al sistema de salud mental. Por otra parte, establecen un marco de justicia social, políticas de identidad y prácticas de apoyo mutuo desde la comunidad. En su conjunto, enfatizan innovaciones metodológicas y una mirada interseccional en la producción de conocimientos. Se concluye que es posible situar la locura como campo de constitución de un actor político y sujeto epistémico. Con base en ello, se formulan posibles líneas de investigación sobre los activismos locos en América Latina.

## Abstract (en)

The following text presents the results of a integrative review whose purpose was to identify and analyze the production of academic literature on mad activism in the field of mental health and its link with the notions of disability and neurodiversity. From searches conducted in May 2023 in the Web of Science, Scopus, and PubMed databases, 52 articles were selected, and thematic content analysis strategies were applied. The results account for various articulations between the concepts addressed, establishing a critical look at the biomedical model in mental health. In the forms of mad activism, the human rights approach, the fight against stigma and its influence on the reform processes of the mental health system become relevant. On the other hand, a framework of social justice, identity policies and practices of mutual support from the community are established. As a whole, they emphasize methodological innovations and an intersectional perspective on the production of knowledge. It is concluded that it is possible to situate madness as a field of constitution of a political actor and epistemic subject. Based on this, possible lines of research on mad activisms in Latin America are formulated.

## INTRODUCCIÓN

En la actualidad, el campo de la salud mental reúne a diversos actores sociales que configuran un ámbito de estudio e intervención. Tomadores de decisión, trabajadores y personas usuarias de salud mental configuran los marcos institucionales de los procesos de atención en este ámbito. En las últimas décadas, el traslado del foco de las instituciones psiquiátricas a los entornos comunitarios ha posicionado en un lugar relevante a las personas que han recibido atención de salud mental^1^. En este contexto, la pluralidad de términos utilizados para señalar a quienes son los destinatarios de los servicios de salud mental -cliente, consumidor/a, usuario/a, experto/a por experiencia, entre otros- refieren la importancia de considerar las valoraciones de los sujetos sobre la atención recibida^2^. Actualmente, la Organización Mundial de la Salud (OMS) y la Organización Panamericana de la Salud (OPS) han adoptado la expresión persona con “experiencia vivida”, denominación que enfatiza la agencia de los sujetos para evaluar los servicios de acuerdo con sus preferencias y expectativas, brindar cuidados y apoyo entre pares, así como desarrollar formas de activismo para promover la inclusión social y el empoderamiento comunitario^3^. 

En términos históricos, las personas con experiencia vivida en el campo de la salud mental irrumpen en el espacio público a fines de la década de 1960 y comienzos de la década de 1970 en EEUU, Canadá y Reino Unido, articulando un movimiento social bajo la identidad política de “expacientes o sobrevivientes de la psiquiatría”^4^. En sus inicios, este movimiento desplegó acciones de protesta en oposición a las prácticas abusivas asociadas al diagnóstico y tratamiento psiquiátrico en espacios asilares caracterizados por la exclusión y el encierro^5^. En una dimensión simbólica y cultural, el movimiento de “expacientes o sobrevivientes de la psiquiatría” favoreció la creación de nuevos sentidos en torno a las diferencias mentales, desarrollando narrativas alternativas a la psiquiatría^6^. De manera ampliada, este movimiento se ha definido como una coalición internacional de organizaciones que trabajan por la defensa de la justicia social y el respeto de los derechos humanos en el campo de la salud mental^7^.

Una de las acciones colectivas contemporáneas del movimiento de “expacientes o sobrevivientes de la psiquiatría” constituye la celebración del “Orgullo Loco” (*Mad Pride*). Esta iniciativa surge en Canadá en 1993 y se ha expandido a diversos lugares del mundo, en función de movilizaciones y campañas públicas que enfatizan el reconocimiento de la locura en el espacio social como una experiencia que merece ser valorada como parte de la diversidad humana^8^. El “Orgullo Loco” como expresión de activismo político en salud mental ha implicado renovadas críticas a la patologización de las diferencias que sustenta el modelo biomédico, así como una denuncia sostenida de las prácticas coercitivas de la psiquiatría^9^. Este movimiento rechaza el lenguaje de la “enfermedad” y el “trastorno mental”, reclama el término “loco” e invierte sus connotaciones negativas, busca un cambio cultural enfatizando una postura de respeto, aprecio y afirmación de la locura como base para una identidad colectiva^10^^,^^11^. De esta manera, el “Orgullo Loco” ha promovido la emergencia de nuevas narrativas que cuestionan la comprensión tradicional de la normalidad y la psicopatología en las disciplinas psi^5^. 

En esta tradición, los estudios locos (*mad studies*) constituyen un campo interdisciplinario de investigación y acción política que busca explorar los conocimientos que han surgido desde el movimiento loco, así como indagar en los imaginarios culturales y sociopolíticos experimentados por miembros de la comunidad loca^12^. Si bien sus orígenes se remontan a Canadá, en los últimos años ha comenzado a emerger como un movimiento internacional, aunque todavía se encuentra en gran parte en el norte global^13^. En específico, los estudios locos valoran y ponen énfasis en el conocimiento en primera persona o experiencial y aunque sus principales referentes son personas usuarias o sobrevivientes de la psiquiatría, no se limita a ellos, involucrando a personas sin experiencia vivida para establecer alianzas de trabajo conjunto y prácticas colaborativas^13^. 

Más allá de los estudios locos, es posible situar ciertos enfoques alternativos y dirigidos por pares que han impulsado procesos de reforma en el campo de la salud mental, como han sido los programas de fortalecimiento (*empowerment*) y recuperación (*recovery*) que tuvieron sus orígenes en el movimiento de “expacientes o sobrevivientes de la psiquiatría”. Desde un posicionamiento crítico, se ha planteado que estos programas han adquirido grados crecientes de institucionalización, clientelismo y subordinación al modelo biomédico^14^. En esta línea, se han realizado revisiones sistemáticas de la literatura sobre narrativas de recuperación en salud mental^15^ y experiencias de personas usuarias de servicios de salud mental^16^, que se han enfocado en la implementación de esta orientación al interior del sistema sanitario y los cambios que ha impulsado en el modelo de atención. 

Frente a ello, en la presente revisión se espera indagar en las concepciones recientes en torno a la locura que surgen en el campo de la salud mental, pero que se posicionan desde una autonomía relativa de los espacios institucionales y se encuentran más cercanas a las dinámicas de politización comunitaria y acción colectiva de las personas con experiencia vivida. Para ello, se tomarán en consideración los referentes contemporáneos del activismo en primera persona que se expresan en el modelo social de la discapacidad y el paradigma de la neurodiversidad. 

En este marco, es relevante aproximarse a las formas actuales de activismo que surgen en el campo de la salud mental desde un posicionamiento crítico hacia el naturalismo y la normatividad del modelo biomédico. Al igual que el movimiento de personas con discapacidad, el Orgullo Loco y los estudios locos dan prioridad a las comprensiones e interpretaciones sociales de la locura. Al respecto, el modelo social de la discapacidad enfatiza el lugar de los procesos históricos y los marcos culturales en la construcción de la discapacidad^17^. A su vez, el paradigma de la neurodiversidad incluye condiciones que se caracterizan por procesos cognitivos y emocionales distintos a la norma, los que configuran una forma reciente de activismo^18^. Este paradigma se desarrolla en oposición a la manera en que la academia y la medicina han definido los grupos particulares de personas en términos de desviaciones patológicas y bajo una noción idealizada de normalidad^19^. En conjunto, el modelo social de la discapacidad y el paradigma de la neurodiversidad se orientan al cuestionamiento de un modelo biomédico que sustenta la patologización de las diferencias, enfatiza la individualización de los problemas sociales, desplaza los determinantes del contexto y prescinde de las narrativas en primera persona en la construcción de conocimientos^12^. 

Por lo tanto, para comprender los debates actuales en torno a estos ámbitos de interés y referencia académica, se plantea la siguiente pregunta de investigación: ¿Qué se ha investigado sobre el activismo loco en el campo de la salud mental, con relación al modelo social de la discapacidad y el paradigma de la neurodiversidad en los últimos años? Para ello, a continuación, se presenta la metodología de revisión integrativa de la literatura con el fin de identificar y analizar lo que se ha producido en este campo en los últimos cinco años, luego se exponen los resultados obtenidos y finalmente se señalan las posibilidades y aperturas para orientar líneas de investigación sobre los activismos locos en América Latina. 

## METODOLOGÍA

La presente revisión integrativa tiene el objetivo de sintetizar y analizar la literatura publicada en los últimos cinco años sobre el activismo loco en el campo de la salud mental, en su vinculación con las nociones de discapacidad y neurodiversidad. Esta revisión combina datos de la literatura teórica y empírica^20^ con el propósito de profundizar en el conocimiento y comprensión de un tema, así como generar una posición o postura informada acerca de este^21^^,^^22^. A partir del análisis de los artículos se plantea identificar los vacíos en la literatura, establecer puentes entre diversas áreas de conocimiento y precisar las necesidades de investigación futura^23^. Los pasos señalados para esta revisión refieren una búsqueda y evaluación de los estudios que serán incluidos, así como el análisis y presentación de resultados^23^. A continuación, se describen las fases que dan cuenta de esta investigación. 

### Fase 1. Procedimiento y estrategia de búsqueda

Para el objetivo de esta revisión se identificó la producción científica sobre el activismo loco en el campo de la salud mental, en su vinculación con las nociones de discapacidad y neurodiversidad entre los años 2018 y 2022. Con este propósito se consultaron las bases de datos Web of Science, Scopus y PubMed en búsquedas realizadas durante el mes de mayo del 2023. Estos repositorios fueron seleccionados por su difusión de producción académica y recopilación de literatura según estándares e indicadores de calidad. La búsqueda bibliográfica incluyó artículos científicos cuyos títulos y resumen se encuentren en idioma inglés, castellano o portugués. En cada una de las búsquedas realizadas, para identificar y clasificar el material se aplicaron los siguientes criterios de inclusión:

1. Artículos de investigaciones empíricas y no empíricas en torno a la locura, la discapacidad y la neurodiversidad.
2. Artículos que aborden el activismo en primera persona, en específico sobre organizaciones de personas usuarias, exusuarias o sobrevivientes de la psiquiatría.
3. Artículos que consideren los planteamientos del movimiento Orgullo Loco y los estudios locos.

**Figura 1:**
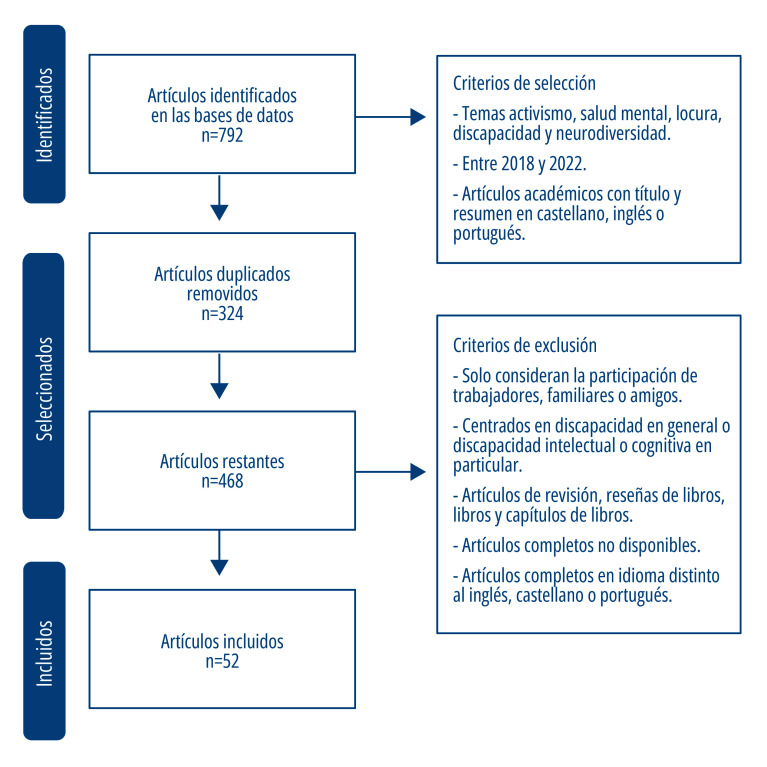
Número de artículos identificados, seleccionados e incluidos en el análisis (mayo, 2023).

Se excluyeron los artículos que solo consideraran la participación de trabajadores, familiares o amigos; que se centraran en la discapacidad en general o discapacidad intelectual o cognitiva en particular; los artículos de revisión, reseñas de libros, libros y capítulos de libros; aquellos artículos no disponibles a texto completo; y artículos que estuvieran en idioma distinto al inglés, castellano o portugués (Figura 1). Para la matriz de búsqueda se utilizaron las siguientes combinaciones de descriptores en idioma inglés, con el fin de tener un alcance más amplio: “*Activism*” AND “*Mental health*”; “*Activism*” AND “*Madness*”; “*Activism*” AND “*Mad*”; “*Activism*” AND “*Neurodiversity*”; “*Activism*” AND “*Mental disability*”.

### Fase 2. Selección de los artículos

A partir de las búsquedas realizadas y mediante los descriptores mencionados anteriormente, se identificaron 792 artículos. Se removieron los artículos duplicados y se obtuvieron 468 artículos. Luego se examinaron los títulos, palabras claves y resúmenes de los artículos, identificando los que cumplían con los criterios previamente definidos para el estudio. Según lo anterior, se seleccionaron 52 artículos para esta revisión, aplicando criterios de inclusión y exclusión (Figura 1). 

Fuente: Elaboración propia.

### Fase 3. Procedimiento de análisis

Con el corpus bibliográfico seleccionado se desarrolló una estrategia de análisis de contenido temático a partir de la generación de códigos, construcción de temas/subtemas e integración de resultados^24^. Para esta revisión la construcción de temas se focalizará en las aproximaciones sobre el activismo loco en el campo de la salud mental y su vinculación con las nociones de discapacidad y neurodiversidad. 

## RESULTADOS

De los artículos que integran la revisión, 48 se encuentran en idioma inglés y cuatro en castellano. El 61% de los artículos fueron publicados por autoras y autores del contexto anglosajón (Reino Unido, 15; EEUU, 10 y Canadá, seis); el 19% de Europa (España, cinco; Alemania, dos; Francia, uno; Suecia, uno y Lituania, uno); el 12% de América Latina (Chile, tres; Brasil, dos y Argentina, uno); y el 6% de África (Uganda, dos y Ghana, uno), el 1% de Oceanía (Australia uno) y 1% del Medio Oriente (Israel, uno). 

Por otra parte, del total de los artículos, 26 corresponden a artículos teóricos y 26 son artículos empíricos. De los artículos teóricos, dos se inscriben en el ámbito disciplinar de la psicología y cuatro forman parte de otras disciplinas de las ciencias sociales. A su vez, tres artículos se inscriben en el ámbito de la ética y la filosofía de la psiquiatría y cinco en el campo de la salud mental global. Los otros textos teóricos, se inscriben en perspectivas interdisciplinares como son los estudios de la discapacidad (cuatro), estudios feministas (tres), estudios locos (dos), estudios trans (dos) y estudios negros (uno). De los artículos empíricos, 19 investigaciones se desarrollaron desde una aproximación cualitativa, cinco corresponden a estudios históricos y dos utilizaron metodología cuantitativa. 

Los resultados se organizan en torno a cuatro temas: 1) debates sobre la producción de conocimiento y la autoridad epistémica en salud mental, que da cuenta de las aproximaciones críticas hacia el modelo biomédico en la articulación entre academia y activismo; 2) enfoque de derechos humanos, lucha contra el estigma y reformas al sistema de salud mental, que refiere a las iniciativas que se inscriben en el ámbito del Estado y las políticas públicas para impulsar cambios en la comprensión y abordaje de la locura, bajo las orientaciones de la discapacidad y la neurodiversidad; 3) marco de justicia social, políticas de identidad y prácticas de apoyo mutuo desde la comunidad, que involucra las experiencias que enfatizan la transformación de las estructuras y sistemas de poder, a partir de la asociación colectiva bajo los principios de la autonomía y la autogestión; 4) innovaciones metodológicas y aproximaciones interseccionales en torno a la locura, que reúne enfoques teóricos y prácticos que orientan la producción de conocimiento para comprender y desafiar las dinámicas de poder, privilegio y opresión en nuestra sociedad. Los ejes temáticos descritos no representan en su totalidad el desarrollo reciente de los activismos locos, sino que dan cuenta de una aproximación situada y construida a partir de la revisión integrativa realizada. 

## RESULTADOS

### Debates sobre la producción de conocimiento y la autoridad epistémica en salud mental

Un primer campo de la literatura describe el desarrollo de aproximaciones epistemológicas que surgen desde el activismo en primera persona y que se distancian de los discursos reduccionistas y patologizadores del paradigma biomédico dominante. En esta línea, se desarrollan enfoques que ponen en cuestionamiento el naturalismo de los trastornos mentales y van más allá de las nociones habituales de desorden, enfermedad, angustia y disfunción para comprender la locura^25^. Al respecto, para el movimiento de personas usuarias o sobrevivientes de la psiquiatría adquieren relevancia los conocimientos subyugados que aportan una mirada positiva en torno a la locura, con base en la elaboración de nuevas narrativas centradas en la experiencia vivida^26^^,^^27^. Si bien el conocimiento experiencial juega un papel importante para el movimiento de personas usuarias o sobrevivientes de la psiquiatría, algunas aproximaciones lo consideran como complementario al conocimiento médico y experto establecido^28^. 

En cualquier caso, lo que se enfatiza es que los actores sociales designados como locos pueden producir conocimiento. Esta aproximación es novedosa en la medida que las personas locas son imaginadas como carentes de racionalidad e incapaces de generar conocimiento; están sujetos a una forma de “injusticia epistémica”^29^. Este concepto, define el proceso de rechazo y desplazamiento en que ciertos grupos sociales son marginalizados y se les priva o niega su capacidad para producir conocimiento. Esta restricción del estatus epistémico en el campo de la salud mental implica una subordinación a la autoridad psiquiátrica cuyo discurso de legitimación es la neutralidad científica^26^. Así, ciertas personas o grupos se permiten hablar y escribir sobre las personas locas porque se supone que no pueden hablar y escribir por ellas mismas^29^. Una de las consecuencias potencialmente dañinas de este silenciamiento es que conduce al descrédito del conocimiento experiencial. Esta “injusticia epistémica” propiciada por la psiquiatría se asocia a las violaciones de derechos humanos y las prácticas coercitivas en la atención de salud mental frente a las cuales las voces de las personas “locas” no son escuchadas^30^. Frente a ello, valorar el conocimiento producido por personas subyugadas por motivos de su competencia epistémica implica perturbar el privilegio dado a algunos “expertos” cuyas intervenciones pueden ser abusivas o perjudiciales^29^. Por otra parte, se subraya que las personas que han experimentado la locura ya han sido ampliamente estudiadas desde una posición de poder, por lo tanto, se requiere un cambio epistémico en la construcción del conocimiento: ha llegado el momento de que las personas con experiencia vivida planteen las preguntas e inviertan el microscopio^27^.

En esta línea, un aspecto relevante para considerar son los acercamientos que han tenido activistas locos con los espacios académicos. En esta tradición, encontramos, por un lado, en Inglaterra a la investigación dirigida por personas usuarias o sobrevivientes de la psiquiatría (*user/survivor research*) que otorga un lugar destacado a las personas locas en la conducción de proyectos de investigación y en la generación de conocimientos^31^^,^^32^. En específico, la investigación dirigida por personas usuarias o sobrevivientes de la psiquiatría busca demostrar la capacidad generativa en la creación de conocimiento por parte de las personas “locas”, sujetos que ahora pueden ser reconocidos como capaces de narrar historias sobre sus propias vidas, pero a los que no se creía que eran capaces de hacerlo^29^. Por otro lado, en Canadá, los estudios locos (*mad studies*) se inspiran en las formas de saber, ser y hacer de las personas locas, y surgen como resultado de iniciativas activistas que han establecido sus raíces en la academia, favoreciendo la narración de aprendizajes y la construcción de herramientas pedagógicas^33^^,^^34^. En particular, el emergente campo transdisciplinario de los estudios locos se relaciona con las experiencias, luchas y análisis de aquellos sujetos que han sido patologizados y violentados por el sistema psiquiátrico^35^.

En su conjunto, ambas iniciativas se orientan a evidenciar las oportunidades, barreras y aperturas para producir conocimientos desde la experiencia en primera persona en los entornos universitarios, así como las alternativas para desarrollar redes de poder y conocimiento colectivo dentro y más allá de los espacios académicos. Estas aproximaciones implican una transformación de las prácticas pedagógicas segregadoras, la inserción de conocimientos locos en el currículo y la despatologización de los sujetos educativos^26^. Del mismo modo, estimulan la conformación de asociaciones de estudiantes locos, que cultivan el apoyo entre pares y desarrollan alianzas para contribuir al cambio institucional^33^. A su vez, los conocimientos locos permiten desafiar los estrechos límites académicos, permitiendo la construcción de comunidades de ayuda mutua dentro y fuera de las aulas, favoreciendo la investigación contra el estigma y la justicia social en los entornos universitarios^34^.

De esta manera, la investigación dirigida por personas usuarias o sobrevivientes de la psiquiatría y los estudios locos dan cuenta de los saberes que nacen de la experiencia vivida en salud mental. Con base en ello, han alterado las dinámicas de poder o autoridad en el terreno del conocimiento, aportando a los debates epistemológicos en torno a la locura como un término complejo y controvertido, con un significado polisémico que, sin embargo, no se circunscribe a un territorio exclusivamente médico. 

### Enfoque de derechos humanos, lucha contra el estigma y reformas al sistema de salud mental

Un segundo campo de la literatura da cuenta de los alcances del activismo en primera persona en el desarrollo de un enfoque de derechos en el ámbito de las políticas públicas y las contribuciones de la locura, el paradigma de la neurodiversidad y el modelo social de la discapacidad en la actualización del modelo de atención de salud mental.

Al respecto, uno de los ámbitos de influencia más relevantes refiere al movimiento de la neurodiversidad, que sostiene que el autismo y otras condiciones neurodivergentes no son trastornos y que representan formas valiosas, naturales y/o normales de la variación neurocognitiva humana^36^. El activismo neurodiverso establece que las diferencias en el cerebro y el comportamiento se encuentran en un espectro no patológico, por lo tanto, cuestionan las definiciones convencionales del autismo como un déficit de funcionamiento^37^^,^^38^. De esta manera, entre los objetivos del movimiento de la neurodiversidad, se encuentran el respeto y la igualdad de derechos, recursos para el apoyo y ajustes razonables, oportunidades educativas y laborales, mejores opciones de tratamiento, eliminación del estigma y un mayor papel de las voces autistas en las decisiones que los afectan individual y colectivamente^36^. En este marco, el movimiento de la neurodiversidad enriquece y amplía una perspectiva de derechos en el campo de la salud mental. 

A su vez, otras publicaciones plantean que el desarrollo de modelos de intervención sustentados en los derechos humanos permite mejorar las relaciones terapéuticas, en especial, los cuidados de enfermería en las unidades de internación psiquiátrica^39^, así como favorecer una regulación sustentada en estándares internacionales sobre el uso de ciertos procedimientos psiquiátricos invasivos e irreversibles, como es el electroshock o la terapia electroconvulsiva^40^. 

Apropiándose de una perspectiva de derechos humanos, el movimiento por la salud mental global o *Movement for Global Mental Health* (MGMH) se ha orientado a crear conciencia sobre la escasez de servicios de salud mental, particularmente, en países de ingresos bajos y medios, enfatizando la importancia de escuchar la voz de las personas con experiencia vivida en los entornos de atención^41^. Del mismo modo, a partir de los lineamientos de la Organización Mundial de la Salud (OMS) se ha reforzado la importancia de la participación comunitaria para aumentar el acceso a espacios adecuados de apoyo social, así como para promover el respeto y reconocimiento social de las personas con diagnósticos de salud mental^42^. 

Otro aspecto importante refiere a la lucha contra el estigma. En el ámbito de las comunidades virtuales, las narrativas de las personas con diagnóstico de depresión han permitido ampliar y desarrollar sus propias nociones de empoderamiento del paciente y experiencia en psiquiatría^43^. A su vez, en el caso de las personas con trastornos de la conducta alimentaria, su participación en línea ha contribuido a implementar acciones para la mitigación del estigma, movilizando un cuestionamiento de las normas sociales impuestas que pueden generar resultados negativos para la salud^44^.

Finalmente, el modelo social de la discapacidad ha favorecido un giro histórico en la comprensión del activismo loco. La transición al enfoque de derechos en salud mental se ha asociado a la implementación de la Convención sobre los Derechos de las Personas con Discapacidad (CDPD) de Naciones Unidas^27^. Estas orientaciones para el cambio se observan en las labores de organizaciones no gubernamentales (ONG) en el desarrollo de campañas locales contra el estigma y la reestructuración de los servicios de salud mental en Europa^45^^,^^46^^,^^47^. También se evidencia, en la creciente influencia de prácticas de solidaridad, activismo social y apoyo entre pares para desafiar el estigma, la exclusión social y la coerción en los entornos de salud mental en África^48^^,^^49^^,^^50^. Del mismo modo, el lugar que han adquirido los usuarios de salud mental en las discusiones sobre políticas públicas, así como su participación en los servicios de salud mental y en los grupos de asesoramiento al consumidor en Oceanía^51^. Finalmente, en América Latina la CDPD ha influido en las formas de involucramiento de organizaciones de usuarios y familiares en los procesos de reforma psiquiátrica permitiendo ampliar su ejercicio de ciudadanía, beneficiando su inclusión y participación en las políticas públicas de salud mental con grados diversos de alcances, consecuencias y legitimidad institucional^52^^,^^53^^,^^54^^,^^55^^,^^56^. 

En definitiva, se constata la importancia del lugar de la abogacía y el cabildeo para desafiar el estigma y promover el respeto de derechos en el campo de la salud mental^57^^,^^58^. Sin embargo, cabe precisar que en este ámbito prevalecen formas de activismo loco que enlazan con una agenda política global orientada a reformar los servicios de salud mental, mejorar los entornos de atención a partir de las normas internacionales de derechos humanos, así como favorecer transformaciones que se sustentan en la estructura de poder del Estado. 

### Marco de justicia social, políticas de identidad y prácticas de apoyo mutuo desde la comunidad

En esta tercera dimensión, podemos situar los avances en el respeto y los derechos que se sustentan en la comprensión de la locura como identidad y cultura. En este sentido, encontramos formas de activismo en primera persona contra el sistema psiquiátrico que tienen por objetivo lograr un cambio en la manera en que se percibe la locura en la sociedad, en términos de una reparación simbólica y cultural^59^.

En ese recorrido histórico, diversas políticas de identidad han configurado las formas de activismo loco en salud mental. En la década de 1970, algunas personas activistas se referían a sí mismas como “reclusos psiquiátricos” o “exreclusos” para destacar su encarcelamiento en asilos y otras instituciones psiquiátricas. Sentían que los términos de “paciente” y “expaciente” -que fueron utilizados por otros activistas- no describían de manera exacta sus experiencias^25^. En la década de 1980 surgen nuevas identidades, en torno a la idea del paciente psiquiátrico como “consumidor” o “usuario” de los servicios. A pesar de que estos términos ganaron popularidad y, de hecho, son utilizados hasta el día de hoy, algunas personas activistas las rechazaron debido a que connotaban la idea de libertad de elección de los servicios en un contexto de falta de alternativas e implicaban un grado de aceptación del modelo biomédico^25^. Finalmente, la identidad de “sobreviviente de la psiquiatría” permitía elevar la voz de los “pacientes liberados” y valorar la capacidad de contar sus propias historias de recuperación. Esta denominación prefiguró las nociones de “experiencia vivida” y persona “experta por experiencia” que son comunes en la actualidad^25^. 

El próximo gran cambio en las identidades de salud mental iba a ocurrir durante la década de 1990 con el surgimiento del movimiento Orgullo Loco^25^. Este movimiento desafía el lenguaje de la “enfermedad mental”, cuestiona las normas y valores sociales que lo sustentan, se apropia de un término con resonancias históricas despectivas y reemplaza sus connotaciones negativas con entendimientos más positivos^60^^,^^25^. El Orgullo Loco no está únicamente relacionado con el funcionamiento de las instituciones de salud mental, sino que tiene un objetivo mucho más amplio y ambicioso que es un cambio cultural en la manera en que se comprende la “locura” y se percibe la “normalidad” en nuestra sociedad^25^. Así, este movimiento se aproxima a una forma de activismo vinculado al modelo social de la discapacidad, al enfatizar la necesidad de un cambio social con base en el reconocimiento de la diversidad emocional y conductual en lugar de una corrección médica del comportamiento^60^. 

Del mismo modo, el activismo de las comunidades neurodiversas, que enfatizan el autismo como diferencia, también se aproximan al modelo social de la discapacidad que considera la discapacidad como impuesta por la sociedad a través de actitudes y barreras^37^. En este marco, es posible ubicar expresiones de activismo que pretenden acabar con el dominio biomédico en la comprensión de la locura, buscando revertir -entre otros propósitos- las percepciones negativas en los medios de comunicación como la peligrosidad inherente de personas con condiciones de salud mental^25^. 

En términos políticos, las organizaciones en primera persona en el campo de la salud mental, bajo los principios del apoyo mutuo y la autogestión, poseen una larga historia de lucha que entiende la justicia social como fundamento del bienestar y autonomía de las comunidades, en oposición a las prácticas institucionalizadas de violencia, abandono y patologización^35^. En este sentido, se ha descrito la importancia de las relaciones de cuidado corresponsables y no jerárquicas, así como los beneficios de las acciones de resistencia y movilización comunitaria frente al modelo biomédico^61^^,^^62^. Al respecto, participar en espacios de apoyo mutuo y activismo de salud mental parece favorecer la incorporación del marco conceptual del paradigma de la recuperación en términos de conexión con los demás y con la comunidad; la esperanza y el optimismo sobre el futuro; la construcción de un sentido positivo de la propia identidad; un significado y propósito vital; así como el empoderamiento para tener control sobre la propia vida^63^.

Estos enfoques promueven alternativas de asociatividad y ayuda mutua, favorecen la construcción de proyectos comunitarios que se organizan a través del apoyo entre pares y la autodeterminación de las personas locas y neurodivergentes, creando espacios seguros que respetan las voces de sus participantes, en lugar de imponer un tratamiento^35^. En esta línea, también se han cuestionado los referentes normativos que obligan a las personas trans a aceptar diagnósticos y evaluaciones psiquiátricas para acceder a la atención médica, impulsando un movimiento por la despatologización trans, que tiene entre sus propósitos la eliminación de la clasificación diagnóstica, así como la denuncia de las prácticas de discriminación y violencia transfóbica en la sociedad^64^. A su vez, se ha descrito que al igual que otros grupos marginados, las personas autistas LGBTQIA+ han desarrollado instancias de autorrepresentación como un medio para aumentar la aceptación y comprensión de sus identidades, así como para lograr espacios comunitarios accesibles e inclusivos^65^. Por otra parte, la teoría crítica de la raza y los enfoques interseccionales de la locura, también han permitido cuestionar las prácticas racistas excluyentes dentro de los movimientos locos, enfatizando que la relación entre raza y locura no es simplemente causal o analógica, sino caracterizada por constelaciones complejas que la determinan y constituyen entre sí^35^.

De esta manera, los posicionamientos analizados instauran lógicas abolicionistas de las instituciones opresivas, enfatizan un rechazo de la enfermedad mental como entidad clínica e impugnan el lenguaje patologizador de la psiquiatría. Desde una aproximación crítica, se ha descrito que el modelo biomédico que despolitiza, silencia y, en última instancia, controla en lugar de escuchar y responder amablemente a la locura no puede transformarse en un enfoque basado en los derechos humanos^27^. En síntesis, las dinámicas de activismo loco descritas en este apartado incluyen la defensa de derechos en un marco más amplio de justicia social, sobre la base de alianzas con otros grupos vulnerados y la conexión con otras luchas sociales para ampliar la denuncia de las diversas formas de abuso, desigualdad y opresión hacia la transformación del orden establecido. 

### Innovaciones metodológicas y aproximaciones interseccionales en torno a la locura

En esta última dimensión, es posible situar las formas de investigación activista y reflexiva que articulan las relaciones con la academia y la producción de conocimiento para y desde el movimiento loco^31^. En este marco, cabe examinar las agendas que dan forma a los esfuerzos activistas según solidaridades y afinidades situadas y contingentes^66^. Estos posicionamientos implican un rechazo a la distinción entre teoría y método para aproximarse a los conocimientos prácticos de las comunidades locas^29^. Por ello, enfatizan que el conocimiento colectivo y plural de las personas usuarias o sobrevivientes de la psiquiatría debe convertirse en un recurso central para desarrollar metodologías diferentes a las actuales y resaltan que el propio saber acumulado puede representar una llave emancipatoria para facilitar un cambio de paradigma en el campo de la salud mental^27^.

Al respecto, encontramos experiencias de investigación colaborativa con comunidades negras y otras comunidades racializadas que se identifican o son categorizadas como locas, neurodiversas, sobrevivientes de la psiquiatría o en situación de discapacidad que son particularmente vulnerables al acoso y la violencia policial^35^. Además, debido a que las concepciones convencionales de los cuidados a menudo han excluido a las personas que están encarceladas, vigiladas y castigadas, y han pasado por alto la condición carcelaria y la expansión del funcionamiento punitivo del Estado^35^, es relevante subrayar la articulación de activismos disidentes o no normativos con los estudios de género y los feminismos. 

En el campo de la salud mental, el movimiento feminista hizo visible la angustia de las mujeres, favoreciendo el desarrollo de críticas feministas hacia la atención psiquiátrica convencional^67^. Desde una mirada interseccional, que permite examinar las desigualdades sociales y la discriminación a partir de la naturaleza interconectada de diversas posiciones de opresión y privilegios, es posible comprender las luchas políticas del “feminismo loco”^68^. En este sentido, la interseccionalidad como marco conceptual para comprender las dinámicas del poder permite referirse a las múltiples violencias denunciadas por las mujeres locas a partir de la conciencia de sí mismas y el reconocimiento entre pares. Al respecto, la relación entre locura y feminismo ha contribuido a cuestionar los procesos de psiquiatrización de las mujeres, impugnando la articulación entre cuerdismo y patriarcado como sistemas diferentes de opresión, pero estrechamente vinculados^69^^,^^70^^,^^71^. Asimismo, la reivindicación de la locura como una forma de ser que disiente de la norma se ha asociado a la reclamación del término *queer*, favoreciendo el nexo de la neurodivergencia y las identidades trans en la lucha por la despatologización de las diferencias subjetivas^33^^,^^72^. De esta manera, se enfatiza la importancia de desarrollar métodos y prácticas que permitan contrarrestar la violencia estructural, así como desplegar intervenciones que fomenten modos de cuidado y justicia abolicionistas interseccionales en lugar de la exclusión, el castigo y la criminalización^35^.

Por otra parte, es posible situar innovaciones metodológicas para el estudio de la locura como parte de un posicionamiento de investigación que articula un marco de justicia social con la acción transformadora. En esta línea, cabe mencionar los estudios de los registros audiovisuales del activismo radical en salud mental que implican una forma de hacer cine políticamente comprometido con base en películas militantes que establecen una crítica hacia el reduccionismo biológico y el discurso de la enfermedad mental^73^. Junto a esta interpretación estética de la locura a través del cine, cabe mencionar el lugar de los estudios locos en el desarrollo de metodologías críticas en el campo de la salud mental que cuestionan la pretensión de objetividad y neutralidad del modelo biomédico y favorecen su inscripción en la tradición de la investigación militante en torno a la articulación de teoría y praxis comprometida^74^. 

Especialmente, en el desarrollo de enfoques cualitativos, se menciona el análisis crítico del discurso como enfoque interdisciplinario que permite un acercamiento a la dimensión simbólica de las injusticias y opresiones sociales, además de favorecer la comprensión de conceptos específicos de la locura y enfatizar la interseccionalidad como un antídoto contra el universalismo^29^. A su vez, cabe destacar los alcances de la autoetnografía. Por un lado, en su vinculación con una aproximación feminista para sustentar una pedagogía crítica del duelo como respuesta feminista a las vivencias de la locura asociada a la pérdida de un ser querido^34^. Por otro lado, en su articulación con las terapias de artes creativas, que permiten examinar las limitaciones del modelo de atención de la salud mental al tiempo que expresan enfoques alternativos arraigados en la tradición activista y la organización comunitaria^75^. Finalmente, su aplicación en un enfoque de acción social basado en las artes, facilitando que agrupaciones juveniles pongan en común experiencias personales sobre temas estigmatizados y fortalezcan su crecimiento como líderes comunitarios^76^. 

En síntesis, las aproximaciones teórico-metodológicas examinadas se orientan a desarrollar prácticas horizontales y colaborativas con colectivos y movimientos para oponerse y desafiar el cuerdismo en la sociedad como forma de opresión especifica hacia las personas locas^35^.

## CONCLUSIONES

En los estudios analizados, se observa un predominio de publicaciones realizadas en EEUU, Reino Unido y Canadá, países en los que tuvo origen el movimiento de expacientes o sobrevivientes de la psiquiatría desde la década de 1960 y 1970. Este movimiento es uno de los fenómenos más prometedores en el campo de la salud mental, por su contribución a la defensa de los derechos humanos y la justicia social^61^. En América Latina, el desarrollo incipiente de este movimiento plantea desafíos para su comprensión y estudio en el contexto de los procesos de reforma psiquiátrica y los ciclos de movilización política recientes^77^. 

Los trabajos empíricos y teóricos incluidos en esta revisión permiten comprender la transición de los expacientes o sobrevivientes de la psiquiatría como personas usuarias de servicios de salud mental a activistas locos, en diálogo con las aproximaciones de la neurodiversidad y la discapacidad. La riqueza de estas articulaciones permite conceptualizar la presencia de activismos locos en salud mental, destacando su concepción plural. Si bien la mayoría de los artículos señalan que este actor social a menudo enfrenta barreras y limitaciones para convertirse en un interlocutor válido para el Estado, moldeador efectivo de políticas públicas o protagonista de las reformas al modelo de atención de salud mental, se constituye como un referente del debate público al desplegar nuevos imaginarios socioculturales en torno a la locura.

En la literatura analizada, las iniciativas impulsadas por activistas locos se han caracterizado por utilizar una variedad de estrategias para desvincular la locura del estigma de la “enfermedad mental”, al subvertir los fundamentos epistemológicos de la psiquiatría mientras imbuían innovadores discursos con una dimensión ética y política. En su conjunto, estas apuestas implican un cambio de perspectiva, en términos de dejar de considerar las experiencias de la locura como “síntomas” de un “trastorno” para situar la comprensión de estas vivencias como fenómenos valiosos, relevantes y significativos en el campo sociocultural^25^. 

Según lo descrito, si bien ciertas formas de activismo loco subrayan la importancia del enfoque de derechos en salud mental, al enfatizar la centralidad de la participación y el involucramiento de las comunidades de personas usuarias y exusuarias en los mecanismos de inclusión social, democratización y ejercicio de ciudadanía en el marco del Estado, cabe destacar las iniciativas que se despliegan en las periferias del sistema político y con autonomía relativa de la esfera institucional. En este punto, adquieren relevancia las iniciativas de movilización comunitaria por la justicia social y las prácticas de resistencia contra la patologización que buscan impugnar los procesos de normalización y control en la esfera de la subjetividad^35^. Estas formas de activismo expresan objetivos diferentes a reducir el estigma o mejorar el acceso a los servicios de salud mental ya existentes^69^. Más bien, manifiestan un descontento con la medicalización de crecientes dominios de la realidad y promueven otras lógicas del reconocimiento de la igualdad y la diferencia^25^. 

En este sentido, toman importancia la presencia de enfoques activistas que proponen innovaciones en los procesos de investigación y producción de conocimiento en salud mental^31^. En particular, los estudios locos resaltan las diferencias epistemológicas entre los discursos médicos expertos y los discursos basados en las experiencias en primera persona. Esta perspectiva permite cuestionar los entendimientos convencionales en el campo de la salud mental mediante la creación de aproximaciones alternativas que surgen desde las experiencias individuales y colectivas de la locura^13^. Lo anterior se vincula con los posicionamientos de las subjetividades subalternas que desafían las dinámicas de violencia, coerción y control predominantes en la sociedad para constituirse en protagonistas de los conocimientos y saberes que surgen de sus luchas.

Así, los estudios locos ofrecen una perspectiva de oposición efectiva a la exclusión y marginación de las personas que experimentan la locura en el terreno de la producción de conocimientos tanto dentro como fuera de la academia^78^. Junto con ello, establecen un marco teórico unificador que tiene como objetivo central la crítica de la psiquiatría biomédica y el desarrollo de contradiscursos críticos y radicales^79^. De esta manera, los estudios locos se desenvuelven en un diálogo interdisciplinario con otras aproximaciones críticas como el feminismo interseccional, la poscolonialidad y la teoría queer^69^. Estas articulaciones se dirigen a impugnar las dinámicas de la opresión y la injusticia social que se expresan en el cuerdismo, el colonialismo, el racismo y la violencia de género, entre otras formas de poder inscritas en las relaciones, instituciones y estructuras sociales. 

A partir de estas orientaciones, que sitúan la locura como campo de constitución de un actor político y sujeto epistémico, resulta relevante aproximarse a las dinámicas de los activismos locos en América Latina. Para ello, habría que examinar los eventuales paralelismos descritos en esta revisión, sin desatender las innovaciones y particularidades de la región en un contexto globalizado. Con todo, es posible sostener que las elaboraciones académicas en este ámbito no se encuentran desvinculadas de las luchas sociales. Por lo tanto, esta propuesta se inscribe en las plurales alternativas en curso^80^ orientadas a indagar en los marcos de sentido que articulan el sujeto y la acción política en el ámbito de la salud mental en nuestro continente
